# Insights into Indentation Cracking of 6H-SiC at Elevated Temperatures Using 3D-FIB Tomography and Crystal Plasticity Coupled with Cohesive Zone Modeling

**DOI:** 10.1007/s11837-026-08500-5

**Published:** 2026-06-29

**Authors:** B-S. Li, N. Grilli, J. Liu, P. Karamched, D. E. J. Armstrong

**Affiliations:** 1 Department of Mechanical and Electro-Mechanical Engineering, National Sun Yat-sen University, Kaohsiung, Taiwan; 2 Department of Materials and Optoelectronic Science, National Sun Yat-sen University, Kaohsiung, Taiwan; 3 High-Entropy Materials Centre, National Tsing Hua University, Hsinchu, Taiwan; 4 School of Electrical, Electronic and Mechanical Engineering, Queen’s Building, University of Bristol, University Walk, Bristol, BS8 1TR UK; 5 Department of Engineering Science, University of Oxford, Parks Rd., Oxford, OX1 3PJ UK; 6 Department of Materials Science and Engineering, FAMU-FSU College of Engineering, Florida State University, Tallahassee, FL USA; 7 Department of Materials, University of Oxford, Parks Rd., Oxford, OX1 3PH UK

## Abstract

High-temperature fracture behavior of the 6H-SiC was investigated using a combination of high-temperature nanoindentation, 3D-FIB tomography, and crystal plasticity finite element modeling coupled with cohesive zone modeling (CPFEM-CZM). At temperatures above 400°C, a clear transition of the indentation-induced crack morphology was observed, including the shift from three–fold $\langle 11\overline{2 }0\rangle$ to six–fold $\langle 10\overline{1 }0\rangle$ corner cracks, and the formation of sub-surface lateral cracks. This thermally-activated transition of crack morphology was clearly captured by incorporating the orientation-dependent slip laws and cohesive surface energies into the CPFEM-CZM modeling, after calibration with the experimental load–displacement data. Successful validation of the CPFEM-CZM model allows detailed extraction of the plastic work and cohesive surface energy from individual crack surfaces, and provides a physical- and indentation-based method for measuring fracture toughness from materials with anisotropic plasticity and fracture. The complicated interplay between plasticity and fracture at the nanoscale can be understood mechanistically using such techniques, which aligns well with the theme of this special edition on bridging the length scale effects in mechanical testing. We dedicate this manuscript to Professor Gerberich’s pioneering work in small-scale fracture mechanics, and hope to continue his legacy.

## Introduction

Silicon carbide (SiC) in its 6H form is a promising strong solid for high-temperature structural applications, due to its unique ability to retain excellent mechanical strength and chemical inertness at elevated temperatures.^1^ However, monolithic 6H-SiC is intrinsically brittle, and is therefore often toughened through chemically grown self-fibers, transforming the simple monolithic crystal into a much more complicated engineering composite. SiC can also be reinforced with metal substrates to overcome its intrinsic brittleness, due to its ease of bonding to most metallic materials.^2^ Overall, SiC-based composites have great potentials in replacing metallic structural components used in aeronautical and nuclear industries.^3^ These structural components will be able to operate in more extreme environments (higher temperature and irradiation dose), hence improving the overall operating efficiency. From a safety perspective, the mechanical properties of the composite’s monolithic constituents or even the fiber–matrix interfaces under service condition must be carefully evaluated before putting them into practical applications.^4^

Nanoindentation is a well-established mechanical testing technique for measuring elastic and plastic properties from small volumes of materials.^5^ Fracture toughness can also be measured from nanoindentation test, though with slightly more effort. Harding et al.^6^ used a sharp Berkovich tip for measuring the fracture toughness (*K*_c_) of monolithic brittle materials, and De Boer et al.^7^ used a blunt wedge-shaped tip to measure the influence of a ductile metallic layer on the brittle Si. Despite being successful in measuring fracture toughness values, they require elastic stress analysis from a well-defined crack geometry and dedicated finite-element modeling. For intrinsically brittle materials, the nanoindentation-based toughness generally shows good agreement with conventional macro-fracture tests. However, as the temperature increases, the stress distribution and crack morphology are modified through dislocation motions, making the nanoindentation-based method less valid.^8^ To extend this technique to high-temperature materials and continue Professor Gerberich’s legacy in bridging the gap of fracture testing between length scales, advanced characterization and modeling techniques are required to capture the changes in both stresses and crack morphology from thermally-activated plasticity.

Room-temperature fracture properties of monolithic SiC have been investigated extensively using indentation-based techniques. Henshall and Kitahara performed Vickers and Berkovich indentation on the 6H-SiC $\left(0001\right)$ basal plane,^9,10^ while Jin et al. observed a variation of the hardness with indenter penetration in $\left(0001\right)$ crystals.^11^ No significant difference in the fracture toughness measurements was observed between the two indenter geometries and showed good agreement with macroscopic values (~ 3 MPa m^0.5^). Kunka et al.^12^ used Knoop indentation to investigate the crack anisotropy of 6H-SiC, and found that two cleavage directions, $\langle 11\overline{2 }0\rangle$ and $\langle 10\overline{1 }0\rangle$, existed on the $\left(0001\right)$ basal plane. Fujita et al.^13^ performed high-temperature Knoop indentation on the 6H-SiC. Both hardness and surface crack morphology were found to be strongly dependent on the temperature, showing direct evidence for the thermally-activated plasticity having a significant effect on the fracture behavior. However, the literature about the high-temperature properties of 6H-SiC is limited; in particular, there is a lack of systematic characterization of the fracture toughness.

The crystal plasticity finite element method (CPFEM) is particularly suitable to capture the deformation behavior of ductile single crystals because it accounts for crystallographic orientations and slip mechanisms. CPFEM has been applied to a wide variety of crystal structures, but not extensively to 6H-SiC. Previous work by Pang et al.^14^ conducted CPFEM simulations for SiC, showing that the nanoindent impressions can be reproduced correctly for differently oriented crystals, as imaged using an optical microscope. It was also possible to predict the most active slip systems, compared with dislocation imaging using the etch pit technique. However, fracture was not investigated.

CPFEM has been combined with different damage models, including phase field fracture,^15^ the Gurson–Tvergaard–Needleman model,^16^ the cohesive zone model (CZM)^17^ and the extended finite element method.^18^ Simulating fracture using the CZM is ideal when all the possible fracture surfaces are known a priori, which is the case for SiC single crystals. CZM is based on cohesive elements, which share nodes with the two crack surfaces and allow the modeling of discontinuities in solids. The comparison between coupled CPFEM-CZM can be used to determine the maximum normal stresses for crack initiation on a crystalline plane and the fracture energy,^16^ which are the two main parameters in CZM. These parameters are related to the fracture toughness and to a length scale which represents the crack opening for which cohesive elements exhibit zero stiffness. Plastic behavior is included because plastic deformation is accounted for in the crystalline grains. Thus, the apparent fracture energy, which is much higher than the actual surface energy due to energy dissipation at the crack tip, is also captured with appropriate mesh size choices. The CZM is particularly suitable for modeling fracture anisotropy, as each crystalline plane can be assigned a different fracture energy. It can also be easily used in conjunction with both CPFEM and isotropic plasticity models to decouple the effect of plastic anisotropy and fracture anisotropy. However, the potential of utilizing the microstructural-sensitive CPFEM–CZM framework to assess high-temperature fracture toughness in conjunction with microstructural-specific experiments has not been extensively tested.

In this work, high-temperature nanoindentation up to 700°C was used to measure the mechanical properties of the single-crystal 6H-SiC, the most commonly used 6H polytypes for manufacturing SiC/SiC composites. The indentation-induced crack patterns were investigated using scanning electron microscopy (SEM) and novel 3D focused-ion beam (FIB) tomography. For the first time, full crack morphology as a function of temperature was revealed. An energy-based fracture toughness calculation based on crack surface area will be presented, and the results are compared with indentation-based measurements. At the same time, temperature-dependent coupled CPFEM-CZM simulations are combined with experimental results to comprehensively investigate the fracture behavior of SiC single crystals to determine the fracture energy and toughness as a function of the temperature, and to explore the different fracture behavior of the various crystalline planes. A comparison between CPFEM-CZM and an isotropic plasticity model is used to understand how the stress distribution induced by the single-crystal anisotropy affects fracture nucleation and propagation. The model parameters are first calibrated using experimental force–displacement curves to ensure accuracy in determining the microscale stress state; then, model predictions are directly compared with the experimental tomography data to understand the origin of fracture and to provide an accurate assessment of the fracture energy on the different crystalline planes. The main novelty of the present work is the combination of plasticity models, CZM, and advanced experimental characterization to simultaneously determine elasto-plastic properties, fracture behavior, and the different crack features occurring on the crystalline planes at different temperatures.

## Experimental

### Materials

High-purity single-crystal 6H-SiC wafers (3 x 3 x 0.2 mm) were purchased from PI-KEM (Tamworth, UK). The as-received SiC wafers exhibited a greenish optical transparency. EBSD was performed to confirm that the indentation plane is the $\left(0001\right)$ basal plane. No further sample preparation was conducted prior to high-temperature indentation.

### High-Temperature Nanoindentation

High-temperature nanoindentation was performed using the Micro Materials NanoTest Xtreme (Wrexham, UK). The nanoindenter is placed inside a vacuum chamber, capable of attaining a vacuum level up to 10^−6^ mbar. A well-calibrated new diamond Berkovich tip with heating capability was used. The calibrated polynomial area function had a leading term (*C*_0_) of 24.5, with later terms (*C*_1_* ~ C*_4_) to describe its deviation from a perfect Berkovich geometry.^19^ The SiC wafer was mounted on a heating stage using the OMEGABOND™ 600 thermal conductive cement, and was heated from RT to 200°C, 400°C, 600°C, and 700°C consecutively, using a constant heating rate of 1.6°C/min. After the 700°C indents, the wafer was cooled at a rate of 1.6°C/min and indentations were also performed at the same temperatures during the cooling cycle. Temperature matching was conducted prior to each test by holding the indenter and sample in close proximity (~ 10 *µ*m) for at least 1 h to minimize thermal drift. All the indents were performed on the $\left(0001\right)$ basal plane, using a constant loading/unloading rate of 10 mN/s. To minimize creep relaxation on the shape of the unloading curve,^20^ a hold time of 20 s was incorporated at maximum load.

### 3D-FIB Tomography

3D-FIB tomography was used to analyze the crack morphology beneath the indent. FIB sectioning was conducted using a Zeiss^®^ Crossbeam FIB-SEM instrument with a Ga^+^ ion beam at 30 kV. Before sectioning, a protective layer of platinum was deposited on top of the indent area. A trench was then milled in the front of the deposited layer to a depth of around 20 *μ*m. The final cross-section surface was cleaned using a beam current of 700 pA, which provided adequate image quality to identify the cracks. High-resolution SEM images were then acquired from the cross-section surface at every 25-nm sectioning interval. A stack of 2D SEM images collected during serial sectioning was then reconstructed and visualized using the Avizo^®^ software (Thermo Fisher Scientific, USA) after sequential image acquisition.

### Crystal Plasticity Finite Element Method and Cohesive Zone Modeling

**Fig. 1:**
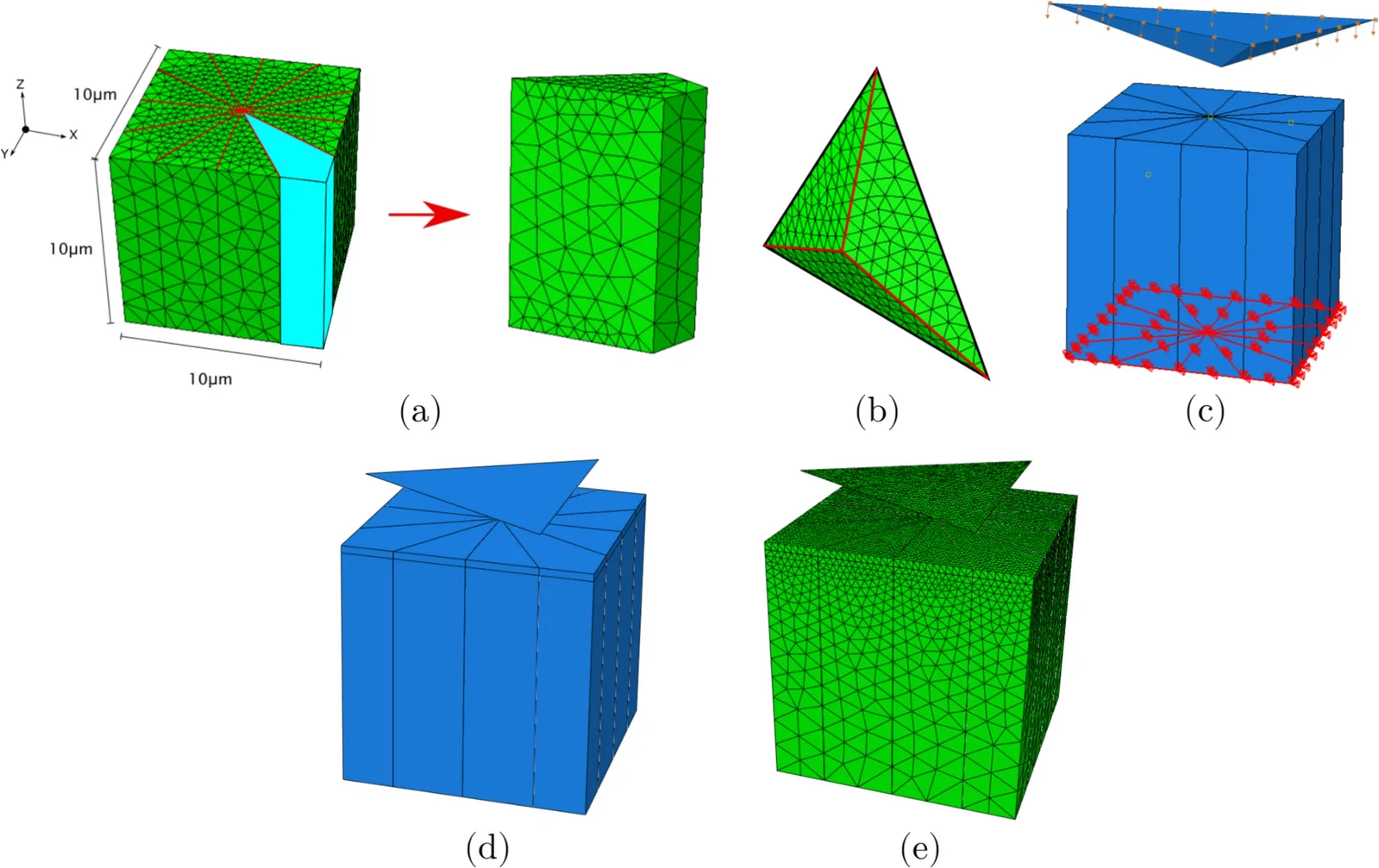
Representative volume and mesh of the coupled CPFEM-CZM simulation: (a) SiC single-crystal substrate for room-temperature simulations, (b) diamond Berkovich indenter, (c) boundary conditions, (d, e) representative volume and mesh with horizontal partition for 700°C simulations.

The representative volume, mesh, and boundary conditions used for the coupled CPFEM-CZM simulations are shown in Fig. 1. The indenter is modeled as a rigid shell so it would not deform throughout indentation, while the geometry of the indenter tip and the angles between the edges are based on a perfect Berkovich indenter geometry, since the experimentally-calibrated area function exhibited a leading term of 24.5. However, it is expected that a blunter tip will require a higher load to nucleate the cracks, causing slight deviation from the modeled load–displacement data.

The representative volume of the SiC substrate is a cube with size 10 *μ*m × 10 *μ*m × 10 *μ*m. The mesh is constituted of 10,692 linear tetrahedral elements with varying element sizes. The element size on the lateral boundary is 1 *μ*m, while the mesh is refined in the center, near the indenter tip, and the smallest element size reaches 0.4 *μ*m, as shown in Fig. 1a and b. The representative volume is partitioned into 12 sections to allow for the inclusion of cohesive elements representing the crystalline planes where fracture can take place. The applied boundary conditions represent the constraint that the substrate imposes on the analyzed small-scale region. Therefore, the displacement on the bottom surface, parallel to the *x*–*y* plane, is blocked in all three degrees of freedom, as shown by the red dots in Fig. 1c. The two lateral surfaces parallel to the* y*–*z* plane have zero displacement along *x*, while the two lateral surfaces parallel to the *x–z* plane have zero displacement along *y*. This is necessary to remove the unrealistic lateral expansion that would be present near the top surface if lateral surfaces are left free. In the model for the simulations at 700℃, an additional horizontal plane with cohesive elements has been added, together with the vertical slices shown in Fig. 1d and e. The depth of the horizontal plane was selected based on the approximate experimentally measured depth of the horizontal crack, which is 0.6 *μ*m below the upper surface. Adding such a horizontal plane with cohesive elements is important from the point of view of accurately determining the stress state in the entire representative volume, as affected by damage on different planes.

**Fig. 2:**
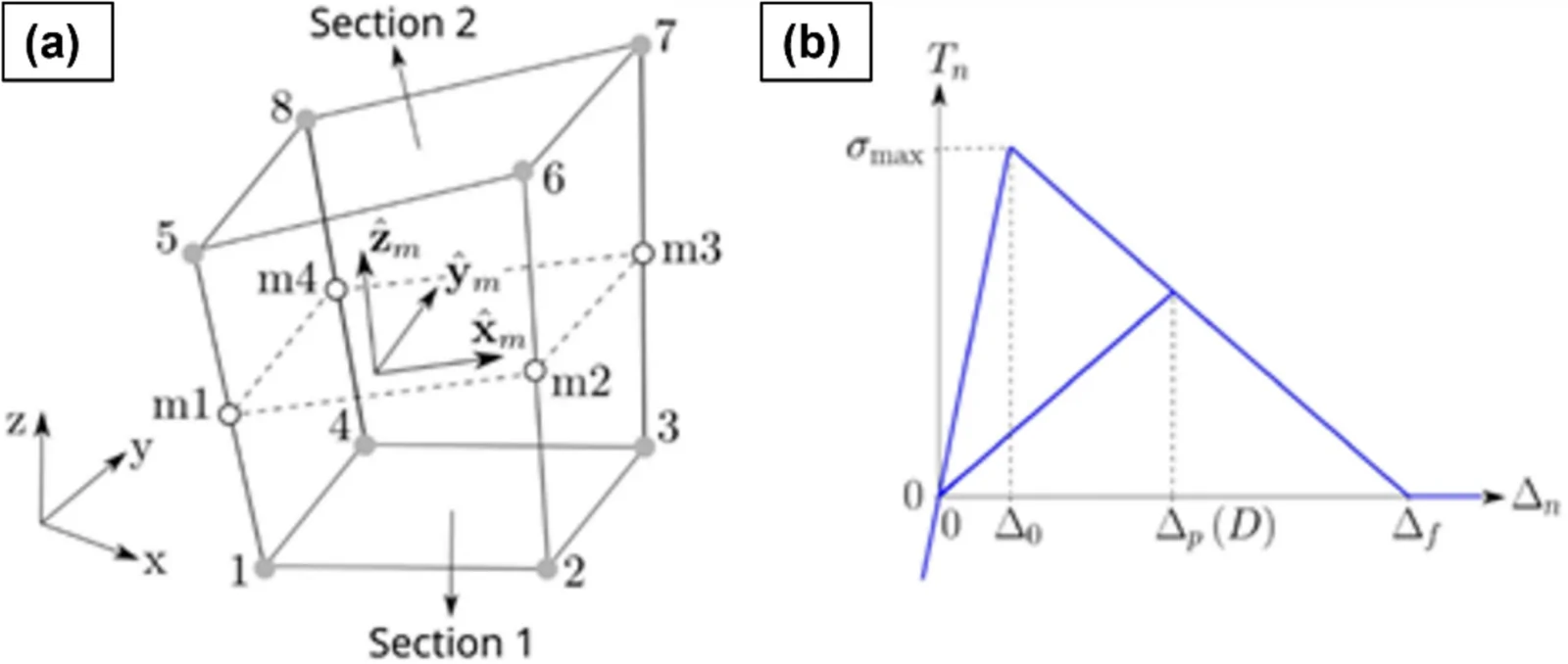
(a) 3D cohesive element with 8 nodes, connecting two sections of the representative volume; (b) bilinear traction–separation law for normal stress.

Contact is used between the indenter and the SiC block; specifically, a surface-to-surface interaction, using small sliding formulation with friction coefficient 0.001 and slip tolerance of 0.005, turns out to have good convergence behavior. The CPFEM model is implemented in Abaqus using the Oxford Crystal Plasticity UMAT,^18^ and the CZM model is based on the Abaqus UEL user element subroutine. Each cohesive element is constituted of 8 nodes, connecting 2 different sections in the representative volume, as shown in Fig. 2a. First-order shape functions are used to describe the normal and shear separation, details of which are described in the work by Grilli et al.^21^ The cohesive law is a bilinear traction–separation law in which the normal traction and shear force increase and decrease linearly with the normal surface opening and shear separation, respectively, as shown in Fig. 2b. Fracture is initiated when the normal traction, *T*_n_, reaches a critical stress, *σ*_max_, with *Δ*_0_ being the separation at which the fracture starts.

Once the crack has been initiated, the normal and shear tractions decrease linearly until the crack has been fully opened when *Δ*_f_ is reached. Stiffness of the cohesive element, which is the initial slope of the traction–separation curve, is prescribed to a value that is much higher than the bulk material stiffness; it is indicated by *K*_*nn*_ for the normal component and *K*_ss_ for the shear component. The representative volume is divided into 12 sections; therefore 12 cohesive interfaces are present. These represent $\langle 11\overline{2 }0\rangle$ and $\langle 10\overline{1 }0\rangle$ directions and can be assigned different fracture energy at different temperatures. The two main CZM model parameters that have been varied in this study to reproduce the experimental results are σ_max_ and *Δ*_f_.

The CPFEM model uses the hexagonal crystal structure and includes 18 slip systems.^22^ Lattice parameters are taken from the literature, with the basal Burgers vector being 0.308 nm and a *c*/*a* ratio of 4.91.^23^ The stiffness tensor of the hexagonal crystal is characterized by 5 independent constants: their ratio has been taken from the literature while the Voigt–Reuss–Hill averaged elastic constant has been matched to the experimental results shown in Table I. The values of the entries of the stiffness tensor are reported in the "Calibration of the Plasticity Models" section. The hardening model is based on geometrically necessary dislocations, calculated through a L^1^ minimization of the total dislocation elastic energy, while satisfying the Kroner–Nye dislocation tensor relationship. The slip resistance, *τ*_*c*_, which is the minimum shear stress on a slip system to activate dislocation motion, grows with the square root of the geometrically necessary dislocation density, while being proportional to the projected shear modulus on the slip system, to the Burgers vector, and to an interaction coefficient, *α*. A constant slip resistance term, *τ*_*c0*_, is also added, which represents multiple effects including the lattice friction and solid-solution strengthening. The slip rate law, determining the plastic shear strain rate on each slip system, is calculated using a thermally-activated Arrhenius-type slip law, in which the slip rate is proportional to the attempt frequency, dislocation density, and an exponential including the activation energy. The stress dependence of the slip rate is described using a hyperbolic sine function, whose argument includes the activation volume and the difference between the resolved shear stress and the slip resistance,* τ*_c_. Details of the numerical implementation are reported in Grilli et al.,^17^ while details of the hardening model are reported in the work by Das et al.^24^ The constant slip resistance, *τ*_c0_, and the interaction coefficient, o, reproduce the experimental and are the two parameters that have been varied in this study to reproduce the load–displacement curves from the nanoindentation experiments.

**Table I:** *E*_s_, H, and *K*_c_ versus temperature for 6H-SiC $\left(0001\right)$ during a heating cycle

Temperature (°C)	*E*_s_ (GPa)	*H* (GPa)	*K*_c_ (MPa m^0.5^)
20	398 ± 4.5	42.5 ± 2.7	1.84 ± 0.15
200	274 ± 16.7	32.8 ± 2.4	2.14 ± 0.19
400	228 ± 10.8	25.3 ± 0.9	N/A
600	198 ± 17.1	20.8 ± 2.1	N/A
700	212 ± 8.6	20.6 ± 1.8	N/A

An isotropic Von Mises plasticity hardening model, coupled with the CZM model, has also been explored to model the bulk behavior and compare with CPFEM. The results are useful to understand how the anisotropy of the plastic deformation in single crystals affect the fracture behavior, effectively decoupling the plastic anisotropy from fracture anisotropy. For the isotropic elasto-plastic model, the value of the elastic modulus at different temperatures is matched to the experimental values reported in Table I, while the Poisson’s ratio used a value of 0.21.^25^ Calibration with the force–displacement curves has provided values for the yield strength as a function of cumulative equivalent plastic strain, as shown in Section "Calibration of the Plasticity Models."

## Results

### High-Temperature Nanoindentation of 6H-SiC

**Fig. 3:**
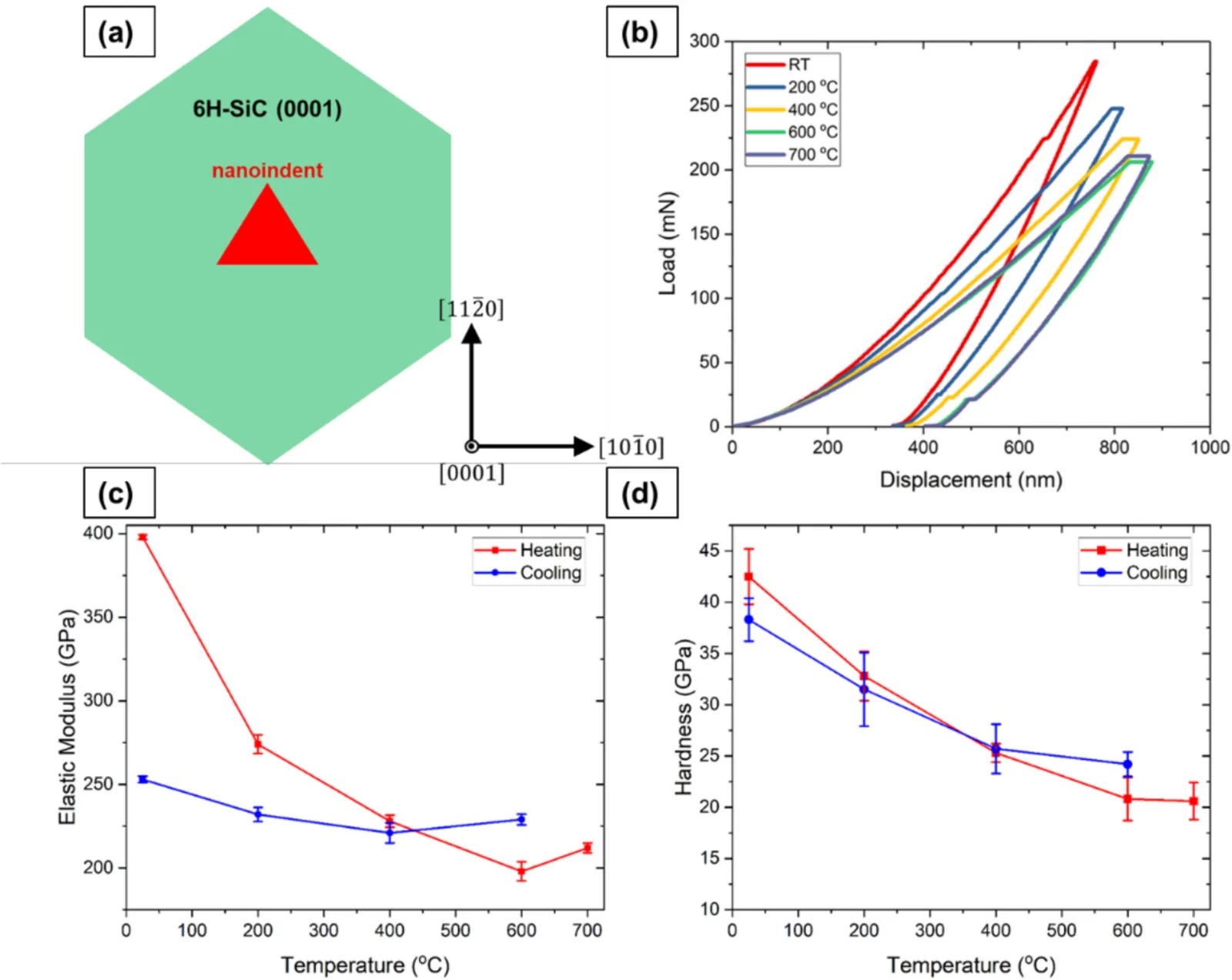
(a) Schematic showing the Berkovich indenter geometry with respect to the wafer orientation, (b) representative load–displacement curves (during heating), (c) sample elastic moduli, and (d) hardness during heating and cooling cycles.

The sample orientation, representative load–displacement curves during the heating cycle, sample elastic moduli (*E*_s_), and hardness (H) up to 700°C are shown in Fig. 3a–d. The reduced moduli (*E*_r_) measured directly from individual load–displacement curves were converted to sample elastic moduli (*E*_s_) using

1 $$ \frac{1}{{E_{{\mathrm{r}}} }} = \frac{{1 - v_{{\mathrm{s}}}^{2} }}{{E_{{\mathrm{s}}} }} + \frac{{1 - v_{{\mathrm{i}}}^{2} }}{{E_{{\mathrm{i}}} }} $$

where the diamond tip modulus (*E*_i_), Poisson’s ratio (*v*_*i*_), and the sample Poisson’s ratio (*v*_s_) were 1141 GPa, 0.07, and 0.21, respectively:^19,24^

E and H at each temperature were average values calculated from nine indents. They showed a gradual decrease from RT (RT) to 600°C, but remained unchanged up to 700°C. Measured elastic moduli remained low throughout the cooling cycle, due to the cracking of mounting cement which increased the overall frame compliance. The total displacement during hold segments were found to increase with temperature, showing that creep deformation is enhanced at elevated temperatures.

**Fig. 4:**
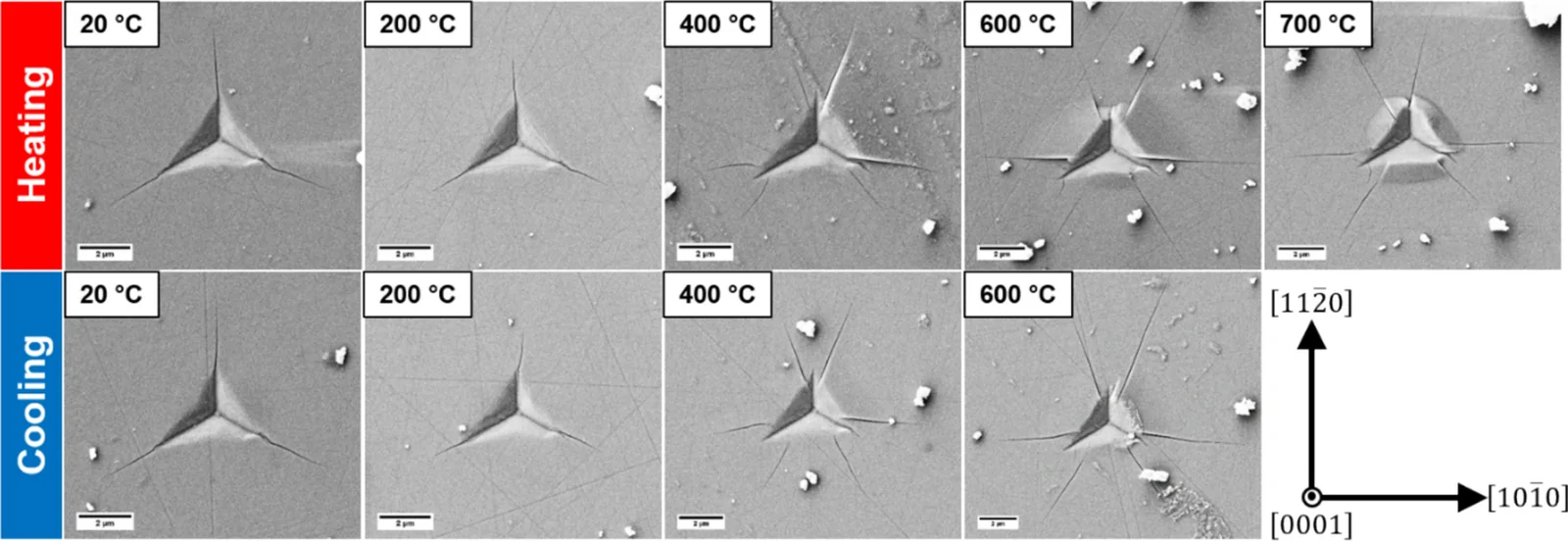
Indentation crack pattern as function of temperature; a change of crack pattern can be observed at temperatures above 400°C.

Figure 4 shows selected SEM images of the nanoindents from RT to 700°C from both heating and cooling cycles. A crack pattern transition was observed at around 400°C, from a single radial crack along the $\langle 11\overline{2 }0\rangle$ direction to two cracks along the $\langle 10\overline{1 }0\rangle$ direction at each indenter corner. The six-fold crack pattern returned to the three-fold crack pattern when cooled below 400°C, confirming that this transition is a thermally-activated process. For indents below 400°C, fracture toughness (*K*_*c*_) values were calculated using the nanoindentation-based Ouchterlony-modified Laugier equation,^26^ provided in:

2 $$ K_{c} = 0.015\frac{{\sqrt {\frac{2}{{1 + \frac{2}{\pi }}}} }}{{\sqrt {\frac{\frac{n}{2}}{{1 + \frac{n}{2\pi }\sin \frac{2\pi }{n}}}} }}\left( \frac{a}{l} \right)^{\frac{1}{2}} \left( {\frac{{E_{s} }}{H}} \right)^{\frac{2}{3}} \frac{P}{{c^{\frac{3}{2}} }} $$

where *n* is the number of radial cracks, *E*_*s*_ and *H* are the sample modulus and hardness, respectively, *P* is the maximum indentation load, *a* is the distance of indenter imprint centroid to the corner, l is the crack length, and c is the sum of *a* and l.

However, the main drawback of the nanoindentation-based method is its strict limitation on the crack geometry; it is only applicable when the crack pattern consists of three radial cracks emanating from indenter corners. For indents ≥ 400°C, there are two cracks at each corner, making this method inapplicable. A summary of *E*_*s*_, *H*, and *K*_*c*_ (only up to 200°C) during the heating cycle is given in Table I. The RT fracture toughness agrees well with literature values also measured via nanoindentation,^27^ and increases by ~ 16% at 200°C. Surface pile-ups were observed for indents above 400°C, suggesting a thermally-activated deformation mechanism.

### 3D-FIB Tomography of the Nanoindents

**Fig. 5:**
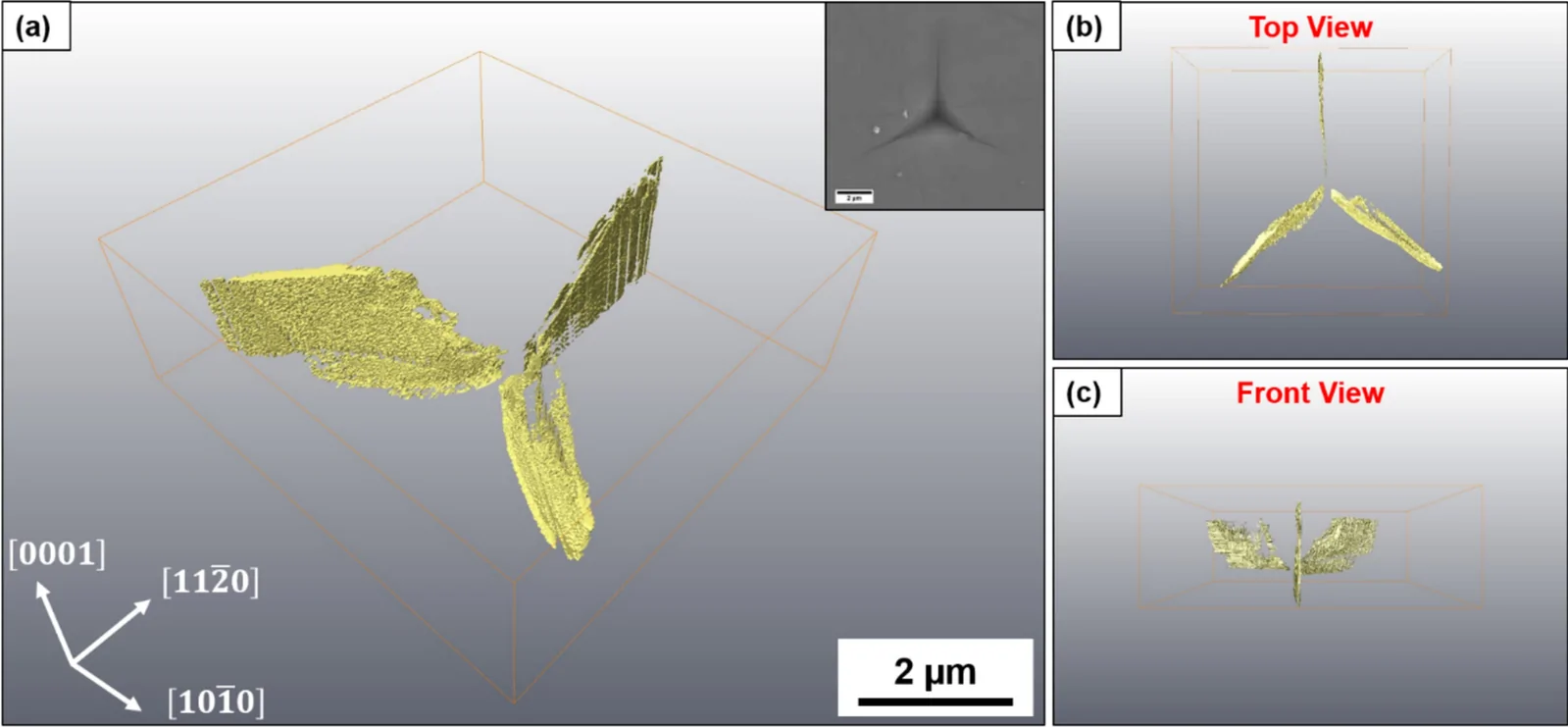
(a) 3D crack morphology of a RT indent, where (b) and (c) are the top and front views, respectively. Only radial cracks emanating from indenter corners are observed.

**Fig. 6:**
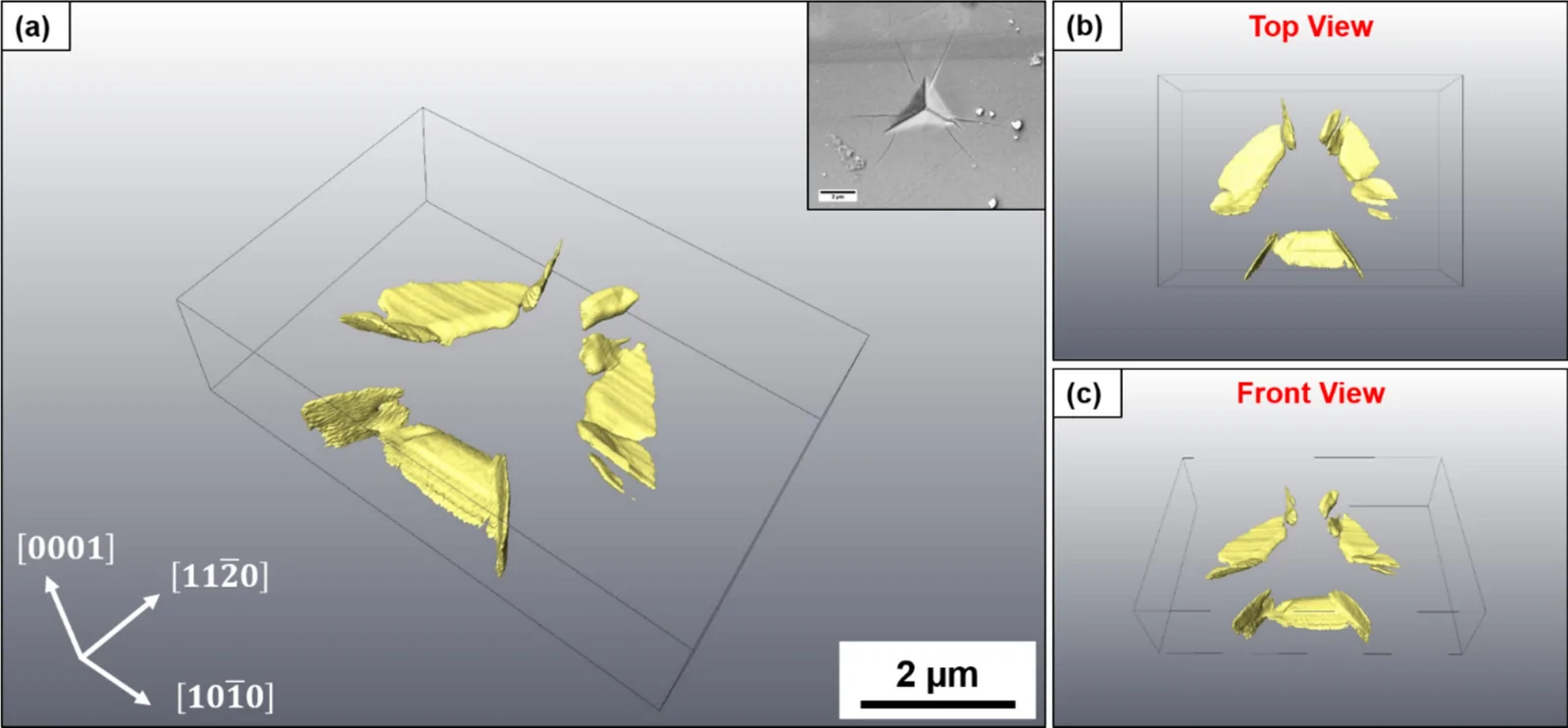
(a) 3D crack morphology of a 700°C indent, where (b) and (c) are the top and front views, respectively. Semi-elliptical lateral cracks are also observed at indenter periphery.

The combination of consecutive FIB slicing and reconstruction is a powerful technique for visualizing the full crack morphology. Figure 5 shows the reconstructed crack morphology from an RT indent. Similar to the surface cracks, three radial cracks along the $\langle 11\overline{2 }0\rangle$ direction were found at each indenter corner. No cracks were found underneath the indenter, due to the compressive stresses beneath a sharp indenter. However, Cuadrado et al.^28^ reported additional lateral cracking in their RT 6H-SiC $\left(0001\right)$ indent, where the radial cracks were connected via laterals crack running parallel to the indenter periphery, which may due to the different in-plane indenter orientation with respect to the indenting plane. Figure 6 shows the reconstructed crack morphology from a 700°C indent. Two shorter $\langle 10\overline{1 }0\rangle$ cracks were found at each indenter corner, with a half-ellipse lateral crack connecting the adjacent corner cracks. Different from the RT crack morphology, lateral cracks were observed beneath the surface of the sample indented at 700°C. These subsurface lateral cracks connected the corner cracks and could not be observed by conventional SEM, which only images the top surface of the sample. The transition of cleavage directions from $\langle 11\overline{2 }0\rangle$ to $\langle 10\overline{1 }0\rangle$ and the additional sub-surface lateral cracks are clearly a thermally-activated process, and will be discussed in terms of dislocation-mediated plasticity.

### Evaluation of Fracture Toughness Using Tomographic Image

**Fig. 7:**
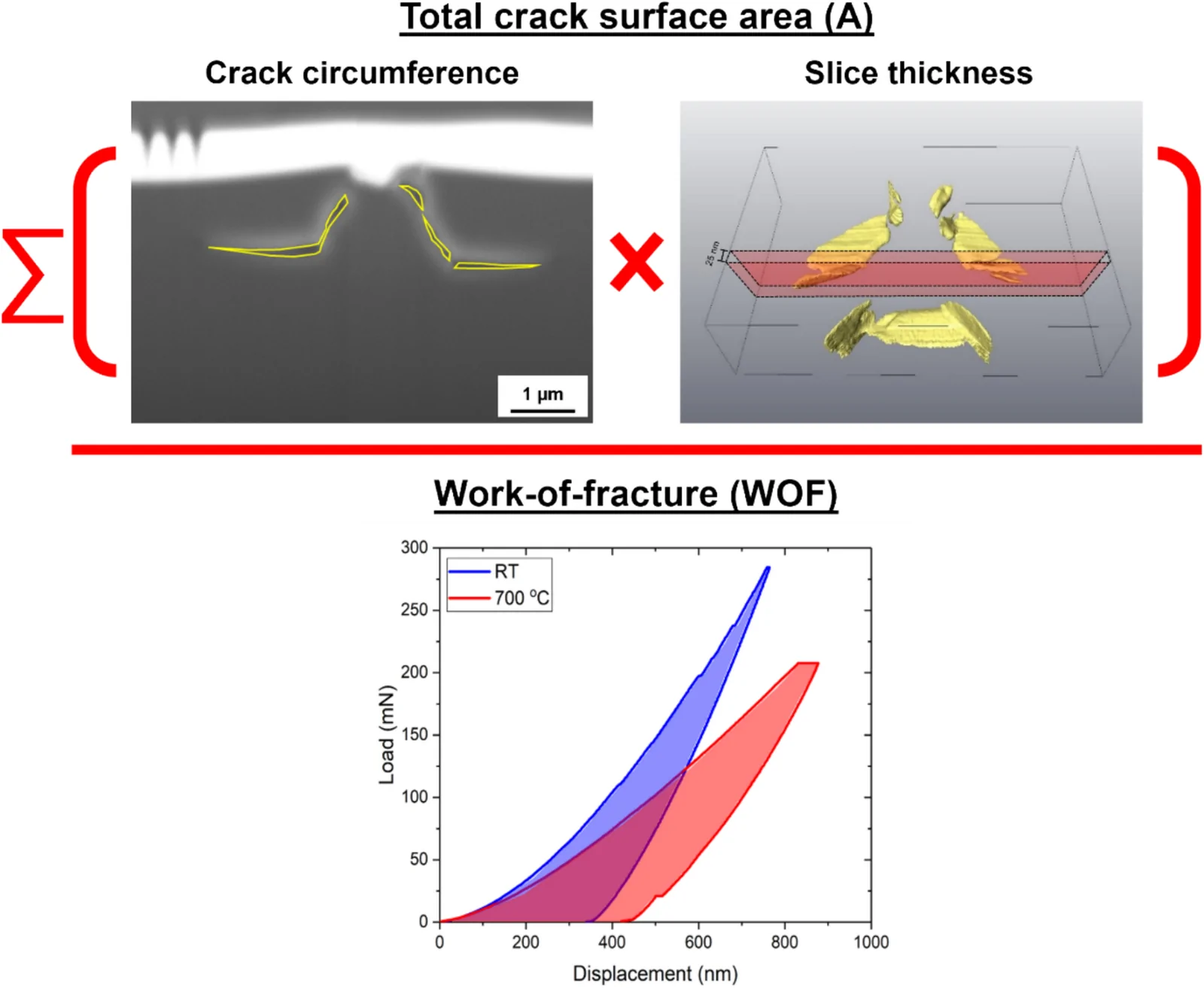
Schematics of the tomography-based fracture toughness calculation. The crack circumference is shown using yellow borderlines, and the slice thickness is shown using the red shaded region. Blue- and red-shaded regions underneath the load–displacement curves indicate the work-of-fracture.

FIB-based tomography provides a novel way of measuring crack surface area. The crack surface area of each FIB slice is calculated using the crack circumference length (yellow line in Fig. 7) multiplying by the slice thickness of 25 nm (red-shaded region in Fig. 7). Hence, the total crack area, *A*, is the sum of crack surface area from all FIB slices. Assuming all the energy provided via the indentation process was used to generate the crack surfaces, then the work-of-fracture (WOF) can be obtained by integrating the area under the indentation load–displacement curve (blue- and red-shaded regions under the load–displacement curve in Fig. 7). Provided with the WOF and *A*, the fracture energy, *G*, can be calculated using Eq. 3. Assuming a plane–strain condition, *G* can be converted to *K*_c_ using Eq. 4:

3 $$G=\frac{WOF}{A}$$

4 $${K}_{c}=\sqrt{\frac{{E}_{s}G}{1-{\nu }^{2}}} $$

where *G* is the fracture energy, *WOF* is the work-of-fracture provided via nanoindentation, *A* is the total crack area, *E*_s_ is the sample elastic modulus, and *ν* is the Poisson’s ratio.

A schematic of this tomography-based fracture toughness method is summarized in Fig. 7, and the calculated crack area, *WOF*, fracture energy, and fracture toughness for both RT and 700°C indents are given in Table II. The main advantage of the tomography-based method is that it does not require any pre-defined crack geometry for stress analysis. Therefore, fracture toughness can be calculated from more complicated crack geometries, including the high-temperature six-fold geometry shown here. Here, the tomography-based fracture toughness calculation is based on an energy approach–mechanical energy provided via nanoindentation consumed solely for creating crack surfaces. Any plastic or time-dependent deformations are neglected. However, there is always energy dissipation through plastic deformation; it is likely that the tomography method will overestimate fracture toughness for materials with significant plasticity.

**Table II:** Summary of tomography results for RT and 700°C indents

Temperature (°C)	Crack area (µm^2^)	WOF (10^−15^ J)	G (J/m^2^)	K_c_ (MPa m^0.5^)
20	20.11	33649	1.67	0.89
700	15.69	43464	2.77	0.93

### Calibration of the Plasticity Models

**Fig. 8:**
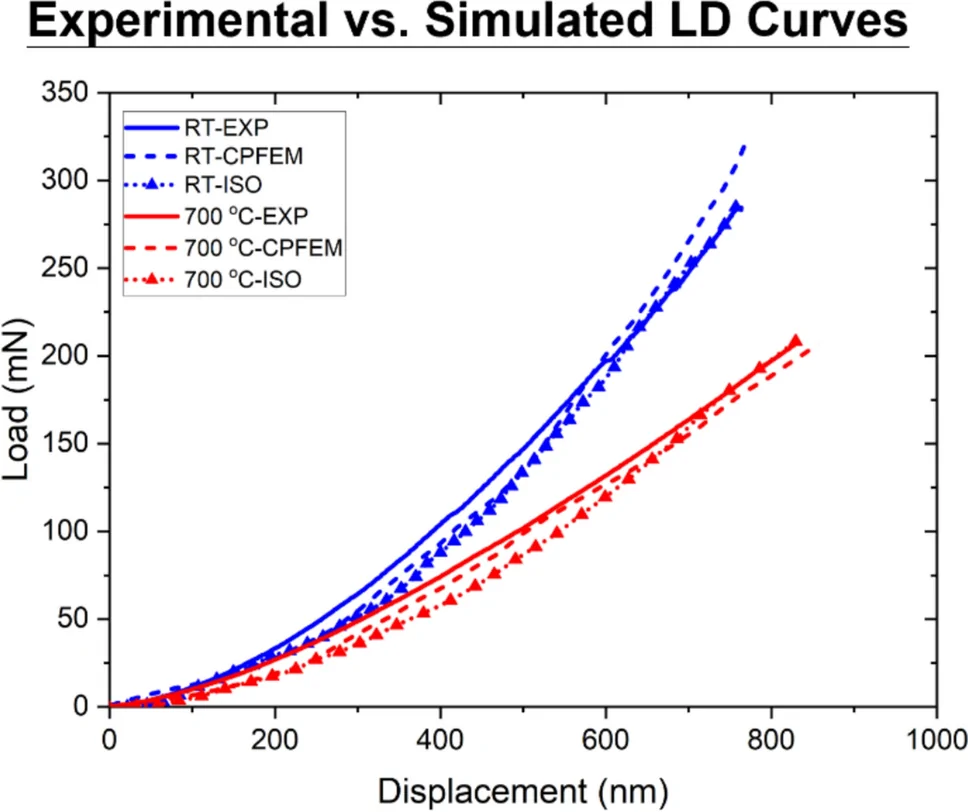
Experimental versus simulated (CPFEM and isotropic hardening modeling) load–displacement curves at RT and 700°C.

Parameters in the plasticity models are calibrated by comparing the simulated results with the experimental force displacement curves during loading. The force is extracted from the simulations by averaging the stress component ${\sigma}_{\left\{zz\right\}}$ over the elements of the indenter on the indenter surface that is parallel to the* x*–*y* plane. The built-in Abaqus rate-independent isotropic hardening model is initially calibrated using data from Grilli et al.,^21^ which are subsequently tuned to minimize the difference with respect to the experimental force–displacement curves at both RT and 700°C. The crystal plasticity model is also calibrated by comparing the simulated and experimental force–displacement curves. The two main parameters of the CPFEM model that have been varied are the constant slip resistance, *τ*_*c0*_, and the interaction coefficient, *α*. The resulting simulated load–displacement curves for the two models at RT and high temperature were validated with experimental curves, as shown in Fig. 8. There is good agreement between the simulated and experimental maximum force, together with the slope of the force–displacement curve. The simulated maximum force remains within 5 mN of the experimental value at RT and within 6 mN at 700°C, while the CPFEM model shows slightly higher slope in some parts of the curve, compared with the isotropic elasto-plastic model.

The calibrated plasticity model parameters for the isotropic elasto-plastic model are set out in Table III with stress for a given plastic strain, which is the format used in the built-in Abaqus rate-independent isotropic hardening model. The constants and calibrated model parameters for the CPFEM model are set out in Table IV. Note that most properties are temperature-dependent, while the assumption that the Burgers vector and interaction coefficient do not change significantly with temperature is made. The slip law is intrinsically temperature-dependent and follows an Arrhenius relationship; therefore, the slip law parameters are assumed temperature-independent.

**Table III:** Calibrated stress–strain curve for the isotropic elasto-plastic model

Stress (GPa)	2	3	4	5	6	7	8	9	10	11	12
Plastic strain (RT)	0.0	0.003	0.009	0.015	0.024	0.036	0.051	0.072	0.085	0.090	0.091
Plastic strain (700°C)	0.0	0.005	0.010	0.012	0.015	0.018	0.023	0.029	0.035	0.037	0.040

**Table IV:** Calibrated parameters for the CPFEM model

Parameter	Room temperature (25°C)	High temperature (700°C)
Elasticity tensor component (*C*_11_)	280 GPa	147 GPa
Elasticity tensor component (*C*_33_)	310 GPa	163 GPa
Elasticity tensor component (*C*_12_)	95 GPa	50 GPa
Elasticity tensor component (*C*_13_)	75 GPa	39 GPa
Elasticity tensor component (*C*_44_)	84 GPa	44 GPa
Elasticity tensor component (*C*_66_)	104 GPa	55 GPa
Voigt–Hill–Reuss shear modulus average (*G*_VHR_)	87 GPa	46 GPa
Burgers vector (basal)	0.308 nm	0.308 nm
Burgers vector (prismatic)	1.512 nm	1.512 nm
Interaction coefficient (*α*)	0.3	0.3
Constant slip resistance (*τ*_*c0*_)	5000 MPa	2530 MPa
Attempt frequency (slip law)	5 × 10^10^ Hz	5 × 10^10^ Hz
Dislocation density (slip law)	1 × 10^12^ m^−2^	1 × 10^12^ m^−2^
Activation energy (slip law)	8.03 × 10^−20^ J	8.03 × 10^−20^ J
Activation volume (slip law)	3.46e−26 m^−3^	3.46e−26 m^−3^

### Fracture Simulation Results

After CPFEM model calibration, the combined CPFEM-CZM model was run to understand the fracture behavior and interpret the experimental results. First, a choice of the characteristic lengths, *Δ*_0_ and *Δ*_f_, in the traction–separation law in Fig. 2 is made to optimize simulation convergence. The choice of *Δ*_0_ = 0.28 nm and *Δ*_f_ = 1.12 nm is made, which respects the Gao–Bower convergence condition,^29^ according to which the energy dissipated during the separation of an interface is larger than the bulk elastic energy released. This avoids the well-known snap-back instability of CZM approaches. Note that these length scales are compatible with the lattice parameter in the SiC lattice, and that they do not directly affect the fracture model calibration and the cohesive surface energy. Specifically, *Δ*_f_ is larger than the basal Burgers vector, thus making the separation at complete failure realistic in terms of lattice spacing between the crystalline planes. The normal stiffness, *K*_nn_ and shear stiffness *K*_ss_, of the traction–separation law are the slopes of the traction–separation curve for an undamaged cohesive element in Fig. 2, in the case which normal and shear deformation is applied, respectively. Their values have to be high enough to avoid artificial compliance that the cohesive elements could introduce. On the other hand, the stiffnesses, *K*_nn_ and *K*_ss_, cannot be too large because of numerical convergence problems. The recommended values must be greater than the Young’s modulus and shear modulus, respectively, as divided by the characteristic bulk element sizes near the cohesive interfaces. In the present work, the values *K*_nn_ = 4.22 × 10^6^ MPa/*μ*m and *K*_ss_ = 4.22 × 10^6^ MPa/*μ*m were chosen. The reference model parameters for the CZM model, which are kept constant in all simulations, are reported in Table V.

**Table V:** Reference parameters of the cohesive zone damage model

Parameter	Value
Normal stiffness (*K*_nn_)	4.22 × 10^6^ MPa/*μ*m
Shear stiffness (*K*_ss_)	1.75 × 10^6^ MPa/*μ*m
Separation for damage initiation (Δ_0_)	0.28 nm
Separation for full damage (Δ_f_)	1.12 nm

**Fig. 9:**
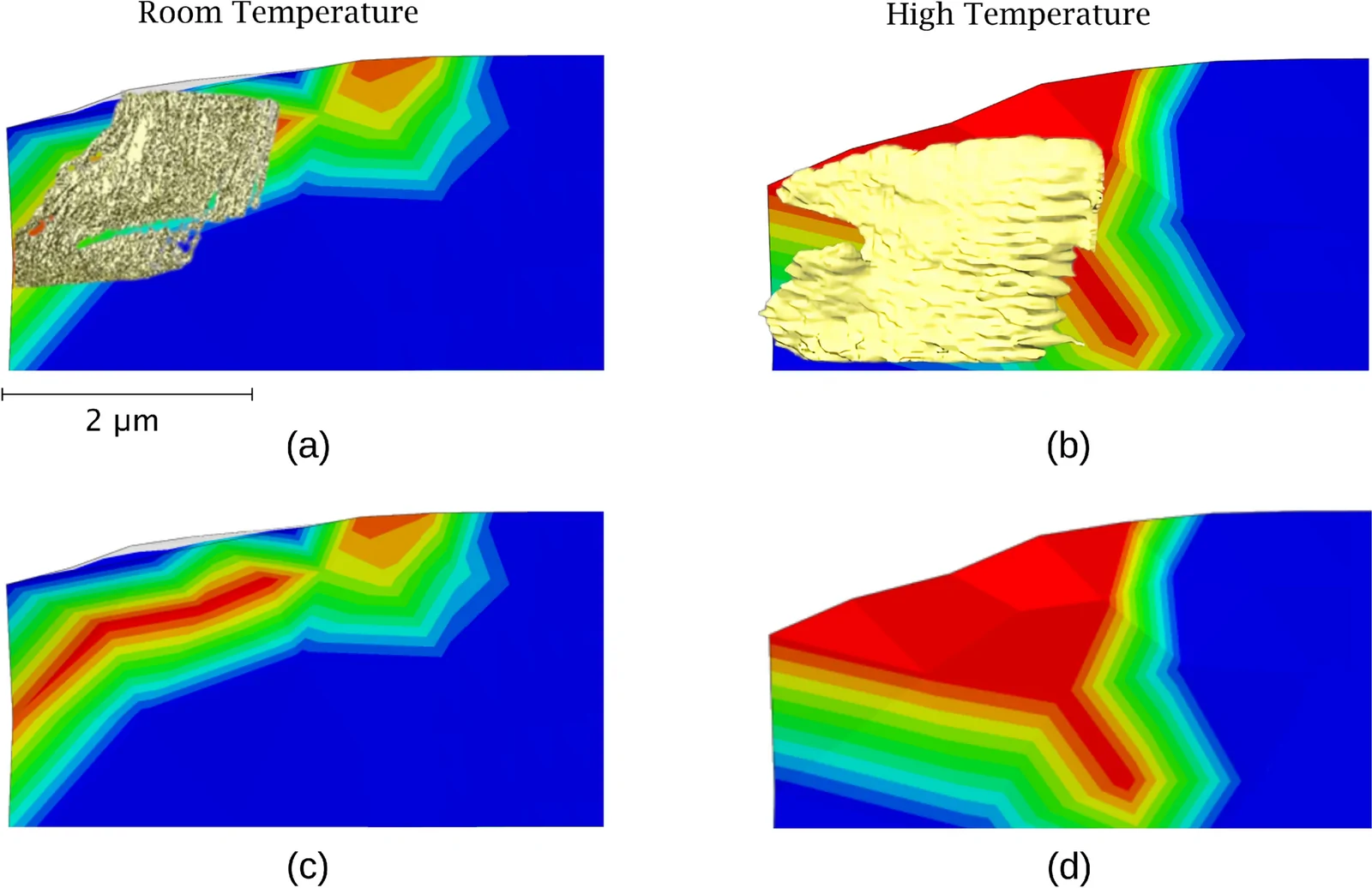
Overlay between simulated (CPFEM-CZM) and experimental damage at (a–c) RT, for an 11–20 crack, and (b–d) 700°C, for a 10–10 crack: the damage field is red in the fully damaged area and blue in the undamaged area.

A parametric analysis is carried out where the maximum stress, *σ*_max_, is varied for different crystalline planes, in steps of 100 MPa, until a good prediction of the crack area, compared to the tomography experiment, is achieved. Specifically, different values of *σ*_max_ for cracks parallel to the $\langle 11\overline{2 }0\rangle$ and $\langle 10\overline{1 }0\rangle$ directions, together with cracks perpendicular to the $\langle 0001\rangle$ direction, are assigned. The simulation results on the various crystalline planes are extracted and analyzed to find the extent of the damaged surface, and the procedure is repeated for both RT and 700°C. This is represented as a color map in Fig. 9: the blue areas are undamaged (damage variable is zero), while the red areas are fully damaged (damage variable is 1). The green areas have an intermediate value of the damage variable, between 0 and 1, which is defined based on how much the slope of the traction–separation curve has degraded after the stress has exceeded *σ*_max_. The parametric analysis aims to match the experimental data in terms of both the qualitative shape of the damaged area and the total crack area in the sample. For this purpose, the experimental tomography image is overlayed on the simulated data, in the same coordinates, which is the yellow feature in Fig. 9.

When the value of *σ*_max_ is decreased on a particular crystalline plane, the damaged area on that plane becomes more extended. For instance, *σ*_max_ = 500 MPa turns out to be too small for all planes at both RT and 700°C, leading to too extensive damage predictions compared with the experiments. On the other hand, values of *σ*_max_ > 3000 MPa lead to very small or absent damage. This monotonic trend allows for the model to be used to predict unique values of *σ*_max_ and corresponding cohesive surface energies. In cases where fracture is not present on a particular plane, for instance $\langle 10\overline{1 }0\rangle$ and $\langle 0001\rangle$ cracks at RT, the predicted *σ*_max_ represents a lower bound.

After the parametric analysis, fracture observables are extracted from the simulations, where the WOF is obtained by calculating the area below the force–displacement curve, as done for the experiment and explained in "Evaluation of Fracture Toughness using Tomographic Image" section. The simulated crack area accounts for the red areas only, as shown in Fig. 9. These post-process results are summarized in Table VI: the WOF prediction is very accurate, within 2% error, thanks to the plastic model parameters calibration, as compared with the experimental force-displacement curve. The crack area is accurately predicted by the CPFEM-CZM model, with an overestimation up to 5% at 700°C, as evident from the slightly higher simulated damage in Fig. 9b. A mesh sensitivity analysis was carried out to understand how the damage predictions change with mesh size and is reported in the appendix. On the other hand, the isotropic elasto-plastic model substantially overestimates the crack area at RT, while it underestimates the crack area at 700°C, as shown by the simulated data in Table VI. The explanation of this behavior requires the detailed analysis of the simulated stress distribution, as explained in the following.

**Table VI:** Comparison of work-of-fracture (*WOF*) and crack area from 3D-tomography and modeling results for RT and 700°C indents

Temperature (°C)	Data source	WOF (10^−15^ J)	Crack Area (µm^2^)
20 (RT)	3D-tomography	33,649	20.11
	CPFEM-CZM	34,008	24.89
	Isotropic elasto-plastic model	33,157	31.13
700	3D-tomography	43,464	15.69
	CPFEM-CZM	42,043	26.65
	Isotropic elasto-plastic model	43,954	18.15

**Fig. 10:**
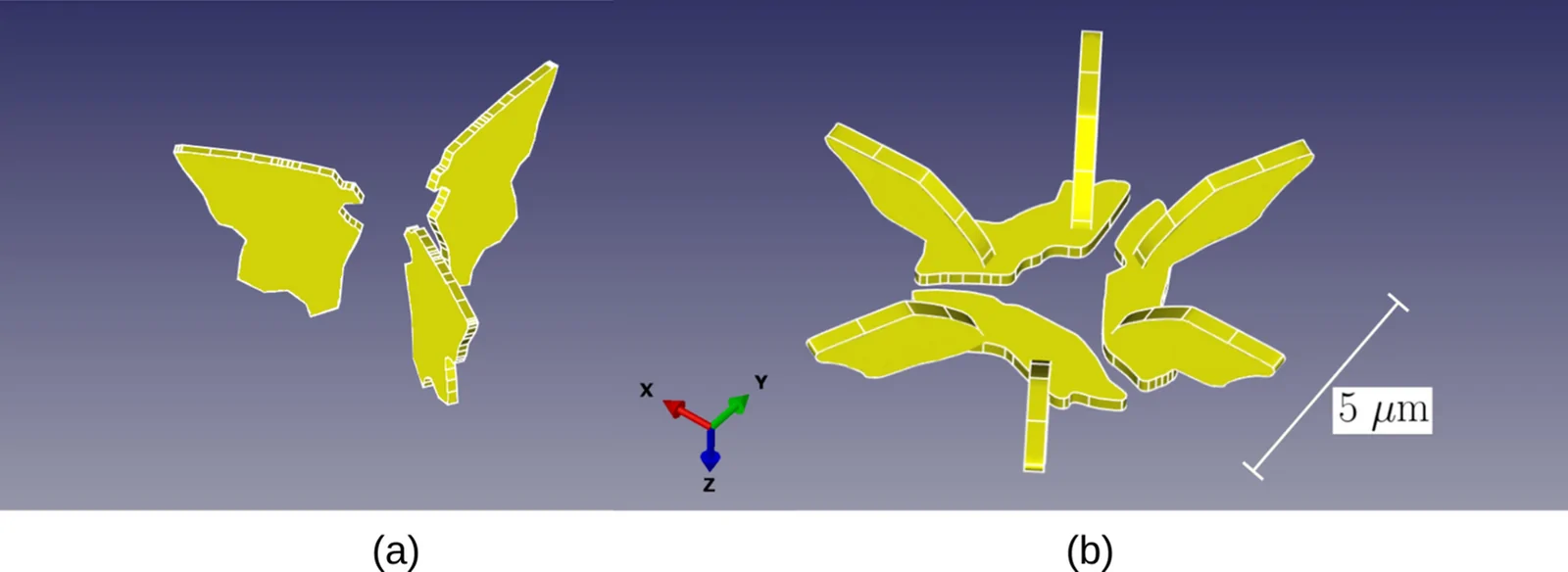
3D reconstructions with optimal fracture parameters (CPFEM-CZM) at (a) RT and (b) 700°C.

A 3D reconstruction of the simulated damage pattern was carried out by using FreeCAD software and by assigning a finite thickness to the cohesive areas that are completely damaged. The results using the CPFEM-CZM model are shown in Fig. 10, where the *y* axis in the simulation corresponds to $\langle 11\overline{2 }0\rangle$ and the *x* axis corresponds to $\langle 10\overline{1 }0\rangle$. This demonstrates the ability of the model to capture the main 3D features of the experimental damage, including the cracks perpendicular to the $\langle 0001\rangle$ axis, compared to Fig. 6.

**Fig. 11:**
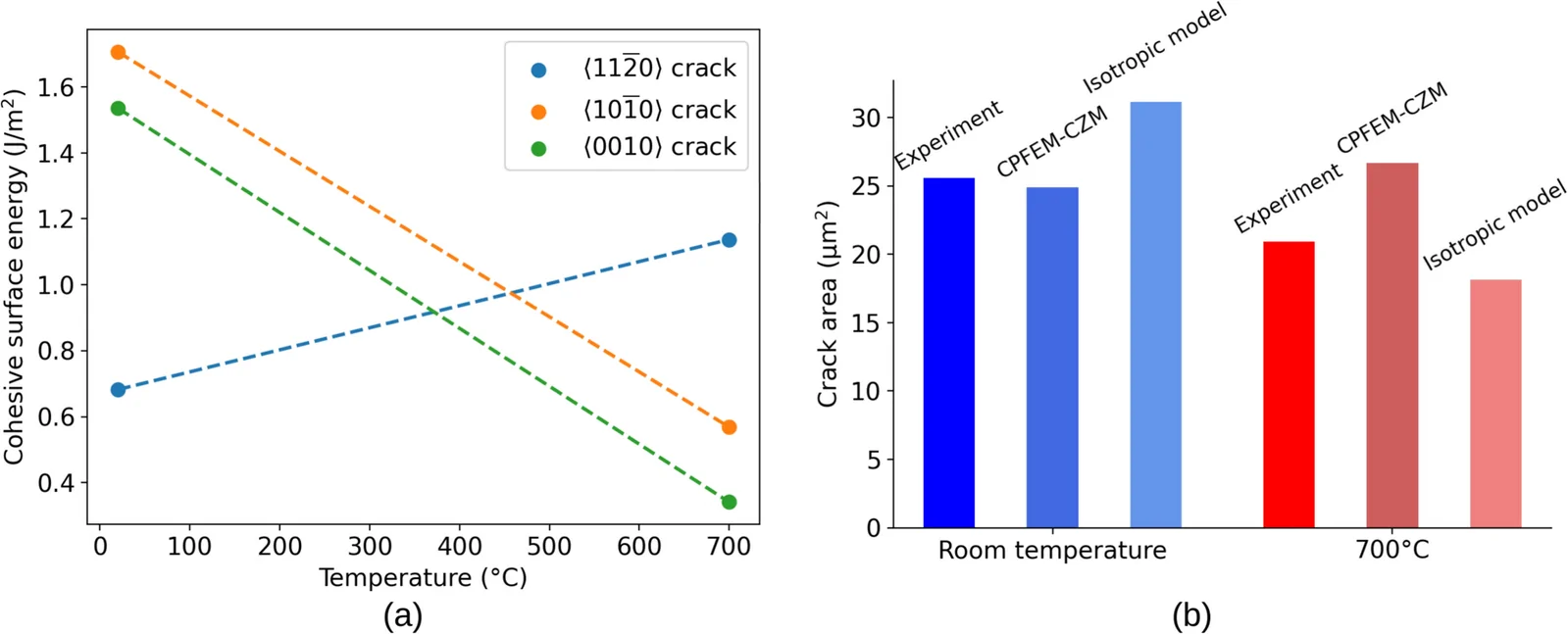
(a) Simulated cohesive surface energy for different crack orientations at RT and 700°C; (b) comparison between experimental and simulated crack area using CPFEM-CZM and isotropic elasto-plastic model.

The simulation results can be used to decouple the energy dissipated due to plastic deformation during the nanoindentation experiment from the energy dissipated by the cohesive surfaces. Specifically, each cohesive surface, once fully damaged, dissipates an energy per unit surface given by the area below the triangle in Fig. 2, which can be quantified as 0.5*σ*_max_*Δ*_*f*_. This quantity will be called cohesive surface energy in the following and can be calculated for each type of crack plane thanks to the parametric analysis determining *σ*_max_. The results are shown in Fig. 11a for the different crack types, and for both RT and 700°C. The $\langle 11\overline{2 }0\rangle$ follows the same trend as the experimentally determined fracture energy, thus increasing at higher temperature. The cohesive surface energy at RT for the $\langle 11\overline{2 }0\rangle$ cracks, which is 0.68 J/m^2^, is about half of the experimentally determined fracture energy, showing that a significant fraction of the energy is dissipated in the bulk because of plastic deformation. At 700°C, the cohesive surface energy of the $\langle 11\overline{2 }0\rangle$ cracks is 1.14 J/m^2^, which is again about half of the experimentally determined fracture energy, thus showing that a larger amount of plastic dissipation takes place due to the increased ductility. Surprisingly, the cohesive surface energy of the $\langle 10\overline{1 }0\rangle$ and $\langle 0001\rangle$ cracks decrease with increasing temperature, demonstrating that the experimentally determined fracture energy is constituted of contributions from different crystallographic planes, whose fracture energies have different temperature trends. This also explains why the $\langle 10\overline{1 }0\rangle$ and $\langle 0001\rangle$ fracture surfaces appear only at higher temperature.

**Fig. 12:**
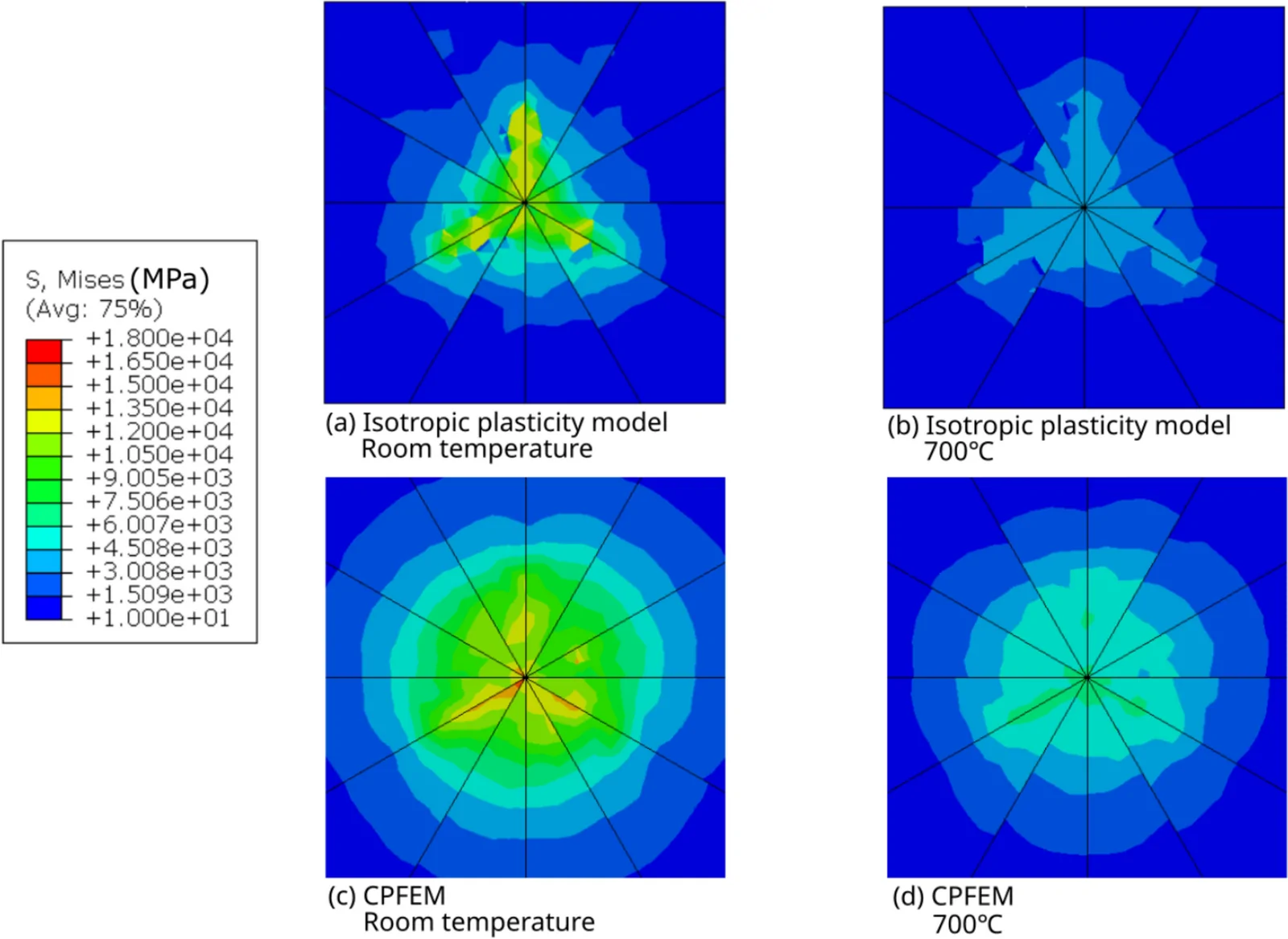
Comparison between the stressfields predicted by the CPFEM and by the isotropic elasto-plastic models: (a) isotropic RT, (b) isotropic 700 °C, (c) CPFEM RT, and (d) CPFEM 700 °C.

The comparison between the isotropic elasto-plastic model and the CPFEM model is used to understand the effect of mechanical anisotropic on crack formation. The predicted crack area for the two models, compared with the experiments, is shown in the histogram in Fig. 11b and interpreted by analyzing the corresponding simulated von Mises stress field at full indenter penetration in Fig. 12. There is a strong difference between the two stress fields predicted by the two models: the isotropic model exhibits stress concentration in a region that has a similar shape to the triangular indenter (Fig. 12a), while the CPFEM model predicts a stress field that decreases radially and is not only concentrated along the $\langle 11\overline{2 }0\rangle$ directions, shown in Fig. 12c. This is due to the anisotropic nature of the deformation in the CPFEM model predictions and explains why the CPFEM-CZM model is substantially more accurate in determining the crack area at RT. The isotropic elasto-plastic model overpredicts the area of the $\langle 11\overline{2 }0\rangle$ cracks at RT, as shown in Fig. 11b, because the stress is excessively concentrated below the three edges of the triangular indenter. At 700°C, the opposite trend holds: the isotropic model underpredicts the crack area because the stress on the $\langle 10\overline{1 }0\rangle$ directions is underestimate; this is because of the inability of the isotropic model to capture the correct anisotropic deformation and resulting stress field, as shown in Fig. 12b. On the other hand, the CPFEM model predicts a more diffuse stress field, with higher stresses along the $\langle 10\overline{1 }0\rangle$ directions, even though these directions are not directly beneath the indenter edges, as shown in Fig. 12d.

## Discussion

### Transition of Crack Pattern at Elevated Temperatures Due to Plastic Anisotropy

Indentation-induced cracks on the $\left(0001\right)$ basal plane were found to cleave along the $\langle 11\overline{2 }0\rangle$ direction at temperatures below 400°C, and to cleave along the $\langle 10\overline{1 }0\rangle$ direction at temperatures above. Previous studies have shown clear evidence of RT plasticity of 6H-SiC using etching pits and transmission electron microscopy investigations.^30,31^ In addition, indentation hardness on the $\left(0001\right)$ basal plane exhibited significant in-plane anisotropy, suggesting that indentation-induced plasticity is dominated by the slip system with the highest Schmid factor. It is now an established fact that plastic deformation on the basal plane of 6H-SiC are mostly accommodated by the $\left\{0001\right\}\langle 11\overline{2 }0\rangle$ and $\left\{0001\right\}\langle 10\overline{1 }0\rangle$ basal slip systems.^13,32^ At elevated temperatures, plastic deformation can also occur via a third prismatic $\left\{ {10\overline{1}0} \right\}11\overline{2}0$ slip system, resulting drop of hardness values. Therefore, the anisotropic nature of each slip system at elevated temperature redistributed the stresses around the indenter, hence changing the crack direction from $\langle 11\overline{2 }0\rangle$ to $\langle 10\overline{1 }0\rangle$. This transition was again found during the cooling cycle, confirming that the redistribution of stresses and hence the crack transition was due to the thermally-activated plastic anisotropy.

### Comparison of Two Cleavage Crack Systems on 6H-SiC Basal Plane

The two cleavage crack directions found on the 6H-SiC basal plane, $\langle 11\overline{2 }0\rangle$ and $\langle 10\overline{1 }0\rangle$, were also investigated using indentation techniques.^9–12^ However, no significant anisotropy was reported, and the fracture toughness of both crack directions was very low, about 2~3 MPa m^0.5^. These results suggest that both directions are an energetically favorable path for crack propagation, and that the active direction is mainly dependent on the stress distributions around the indent, which is the accumulative results of the thermally-activated plasticity.

### Comparison of K_c_ Between Indentation and Tomography Methods

RT fracture toughness calculated using tomography is significantly smaller than values calculated from the indentation-based method (0.79 versus 1.8 MPa m^0.5^). For the 700°C indents, tomography calculates a fracture toughness value of approximately 0.81 MPa m^0.5^, which is similar to the RT tomography-calculated value but again smaller than literature values from the crack system (2.4 MPa m^0.5^). It is known that the two active crack systems on the 6H-SiC basal plane, $\langle 11\overline{2 }0\rangle$ and $\langle 10\overline{1 }0\rangle$, exhibits similar fracture toughness values, which is consistent with tomography calculations. However, one should expect tomography to calculate a larger fracture toughness due to energy dissipation through plastic deformation at elevated temperatures. It is possible that additional crack volumes were introduced during FIB slicing, due to the release of elastic compressive residual stresses from the surrounding matrix, hence resulting in a smaller fracture toughness value.

### Modeling Discussion

This work demonstrates the need to use CPFEM simulations to capture the accurate stress field that is necessary for anisotropic damage predictions. The comparison between CPFEM and the isotropic elasto-plastic model shows that the latter is inadequate for damage predictions and cannot accurately capture the crack area. Specifically, the specific slip activation in the SiC single crystal leads to a more distributed stress field, which is not concentrated just beneath the indenter edges.

Simulations can be used to complement the experimental methodology and to determine which fraction of the experimentally determined WOF is caused by plastic deformation in the single crystal. This is essential to correct the experimentally determined fracture energy, thus providing an accurate value of the fracture toughness for cracks along specific crystalline planes. The CPFEM-CZM simulations provide an essential bridge between force–displacement curve, tomography images, and the determination of the cohesive surface energy of individual crystalline planes. This is not achievable using just experiments, as nanoindentation experiments cannot be easily controlled to selectively induce cracks on a single crystalline plane.

There are a few limitations of this computational technique. First, the boundary conditions also induce some damage away from the indenter center, as shown, for instance, in Fig. 9b. As a result, the agreement is good near the indenter, while some damage is predicted outside this central region. In this work, we have focused on damage in the vicinity of the indenter tip. Second, the potential fracture surfaces need to be prescribed; however, the techniques, such as phase field fracture, could be used to overcome this limitation. Third, if a given crystallographic plane does not fracture, only a lower bound for the cohesive surface energy can be determined; in such cases, atomistic simulations could be useful for complementing CPFEM-CZM and for obtaining more accurate estimates.

## Conclusion

High-temperature nanoindentation combined with FIB tomography were used to investigate indentation-induced cracking of 6H-SiC $\left(0001\right)$ up to 700°C. Both hardness and moduli decrease with increasing temperature, and a transition of crack cleavage direction was found at around 400°C, which are due to the thermally-activated plastic flow on the basal plane. Crack morphology for RT and 700°C indents were reconstructed using consecutive FIB slices, showing three $\langle 11\overline{2 }0\rangle$ radial cracks at RT and six $\langle 10\overline{1 }0\rangle$ corner cracks with extensive lateral cracking at 700°C. A new fracture toughness calculation based on FIB tomography is proposed, which is not limited to specific crack geometry, e.g. radial cracking at indenter corners. Fracture toughness calculated using tomography were significantly smaller than values from indentation. This is in contrast with what was expected, possibly due to the additional cracking from the FIB slicing. Both literature (mostly via Vickers and Knoop indentation) and tomography consistently reported similar fracture toughness for the two cleavage directions. The combination between experimental results and combined CPFEM-CZM simulations allows the fracture energy on individual crystallographic planes to be determined. The results suggest that tomography is a potential method for calculation fracture toughness from nanoindents with more complicated crack geometry.

## Figures and Tables

**Fig. 13:**
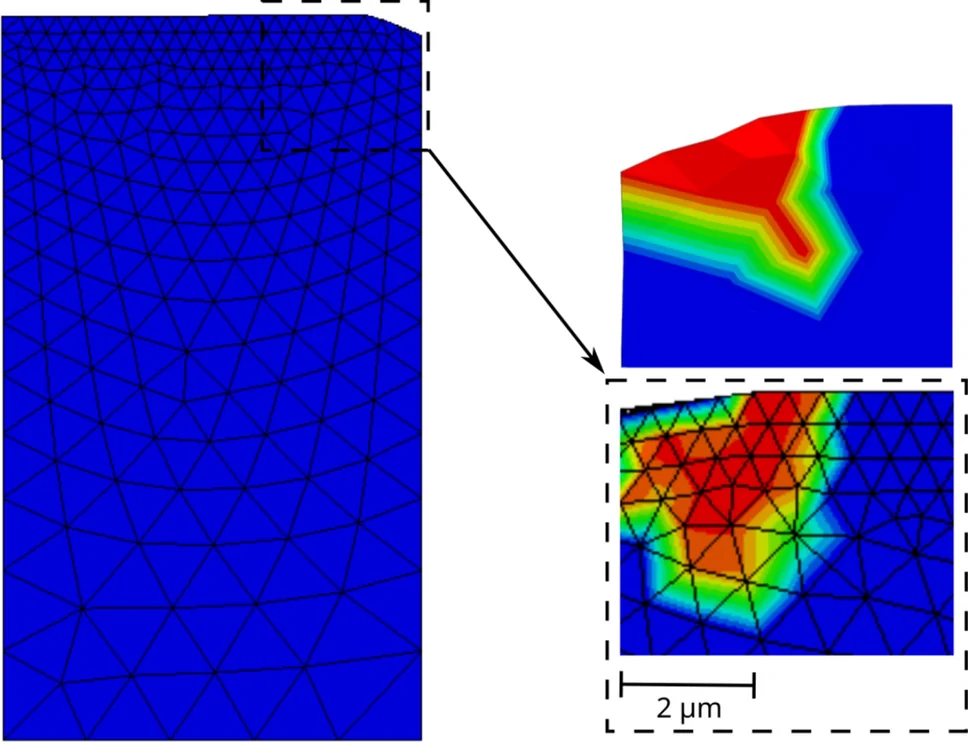
Mesh refinement and damage prediction using two different mesh sizes near the indenter tip.

**Fig. 14:**
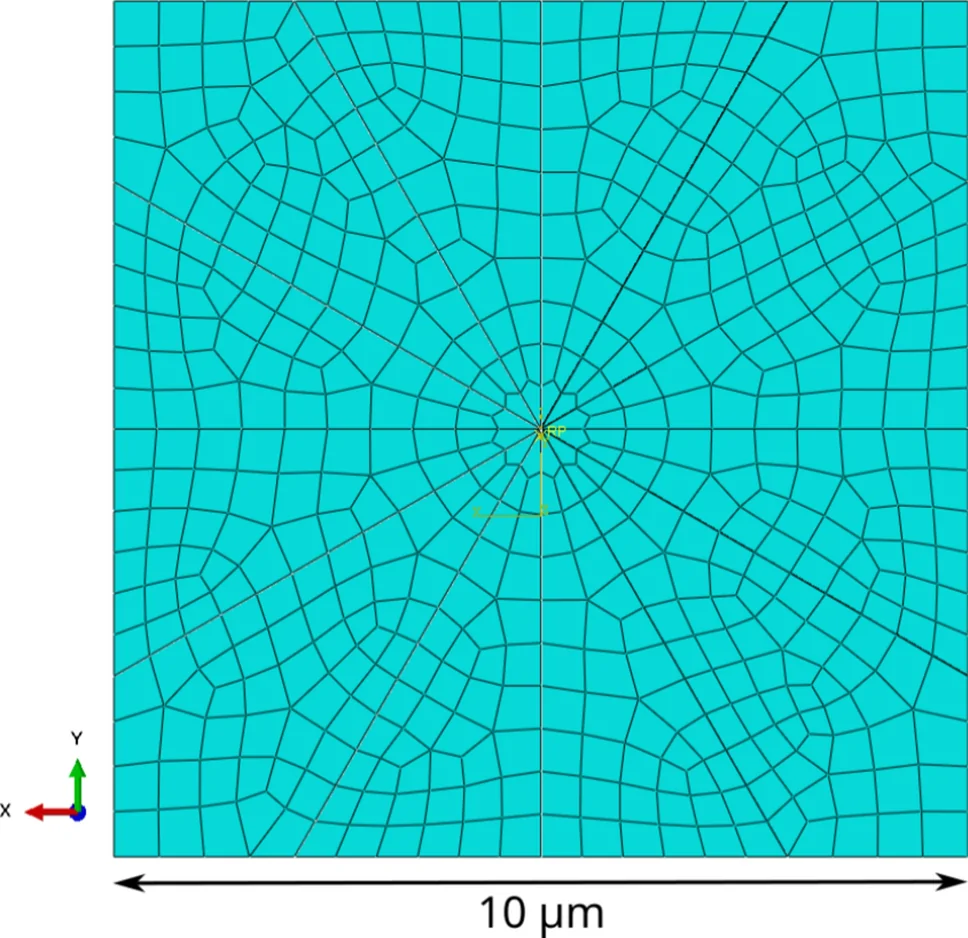
Example of hexahedral mesh used for the simulation study.

## Appendix

A mesh refinement study was carried out to understand the effect of the mesh size near the indenter tip on the fracture predictions. The mesh presented in Fig. 1 has an element size of 0.4 *μ*m near the indenter, while only a single layer of elements at the top surface is refined. Figure 13 shows an example of a refined tetrahedral mesh, where the mesh is refined both along the indentation direction and along the lateral direction, with multiple layers of refined elements near the top surface. Figure 13 also shows the corresponding damage prediction, compared with the coarser mesh used in Fig. 1. The red region represents the fully damaged area, and the two meshes predict a crack length in the lateral direction that is the same within 0.2 *μ*m, demonstrating that the results are not strongly mesh size-sensitive. For computational efficiency, the previously shown results have been obtained with a coarser mesh.

A mesh with hexahedral elements was also generated, as shown in Fig. 14. However, due to the partitioning of the substrate representative volume into 12 slices and the consequent small angle of the corner of each slice near the indentation region, the aspect ratio of a hexahedral element at the indenter tip, which is the ratio of its longest edge to its shortest edge, turns out to be too large compared with finite element analysis recommendation, leading to an inaccurate solution. Because of this, a tetrahedral mesh was used in the results. The element size needs to be larger than the characteristic lengths, Δ_0_ and Δ_f_, in the traction–separation law to avoid an unrealistic representation of the brittle fracture process. Those characteristic lengths are constrained by the Gao–Bower convergence condition discussed. Therefore, the level of refinement proposed is reasonable from this point of view.
